# Immunogenic mapping of potential epitopes from Tc-CTL-1 for the diagnosis of murine toxocariasis

**DOI:** 10.1590/0074-02760240111

**Published:** 2025-03-14

**Authors:** Gabriela Rodrigues e Fonseca, Ana Laura Grossi de Oliveira, Ramayana Morais de Medeiros Brito, Marcelo Andreetta Corral, Richard John Ward, Pâmela Aparecida de Lima, José Bryan da Rocha Rihs, Marcelo Eduardo Cardozo, Paula Keiko Sato, Ricardo Toshio Fujiwara, Sergio Vieira dos Santos, Ronaldo Cesar Borges Gryschek, Susana Angelica Zevallos Lescano

**Affiliations:** 1 Hospital das Clínicas da Faculdade de Medicina da Universidade de São Paulo, Laboratório de Investigação Médica em Imunopatologia da Esquistossomose e Outras Parasitoses, São Paulo, SP, Brasil; 2 Universidade Federal de Minas Gerais, Laboratório de Imunobiologia e Controle de Parasitos, Belo Horizonte, MG, Brasil; 3 Universidade de São Paulo, Faculdade de Filosofia, Ciências e Letras de Ribeirão Preto, Departamento de Química, Ribeirão Preto, SP, Brasil; 4 Hospital das Clínicas da Faculdade de Medicina da Universidade de São Paulo, Laboratório de Investigação Médica em Imunologia, São Paulo, SP, Brasil; 5 Faculdade de Ciências Médicas da Santa Casa de São Paulo, Departamento de Ciências Patológicas, Laboratório de Parasitologia, São Paulo, SP, Brasil

**Keywords:** Toxocara canis, mice, immunodiagnosis, synthetic peptide

## Abstract

**BACKGROUND:**

Toxocariasis is a neglected global zoonosis. The immunological diagnosis has setbacks that hinder further knowledge about its pathology, epidemiology, and public control measures, and lack of financial support and attention prevents innovative research. Although studies on synthetic peptides are common for several infectious pathologies, none evaluated chemically synthetic peptides for toxocariasis diagnosis.

**OBJECTIVE:**

This study aimed to identify potential synthetic peptides from C-type lectin 1 (Tc-CTL-1) from *Toxocara canis*.

**METHODS:**

*In silico* analyses were made by five B-cell peptide prediction programs, 3-D modelling, BLASTp homology analysis, and signal-peptide identification. SPOT-synthesis was used for epitope mapping and assessed by dot-blot. Sera from non-infected and *T. canis*, *Strongyloides venezuelensis*, *Ascaris suum*, or *Schistosoma mansoni*-infected animals were used to assess the peptide’s immunogenicity and cross-reactivity. The selection of potential immunogenic epitopes included the most immunogenic peptides with the least cross-reactivity.

**FINDINGS:**

Fifty-five peptides were selected by *in silico* analysis. Dot-blot showed intense recognition by anti-*Toxocara* IgG and cross-reactivity with *A. suum-*infected mice. Selection criteria identified four epitopes with diagnostic potential.

**MAIN CONCLUSIONS:**

The findings demonstrate that synthetic peptides should be explored for innovation of toxocariasis diagnosis, and suggest the adaptation of dot-blot using the SPOT-synthesis technique as a potential immunodiagnostic platform.

Toxocariasis is a zoonotic parasitic disease caused by the adult and larval stages of *Toxocara* spp. The parasite is the causative agent of neurotoxocariasis, common or covert toxocariasis, visceral and ocular larva migrans. Humans and other animals, such as rodents, serve as paratenic hosts, wherein the larvae do not complete their life cycle and may either migrate through tissues or remain arrested within granulomas.^1^ ^,^ ^2^

Approximately 19% of the world’s population has anti-*Toxocara* antibodies; however, prevalence estimations rely on immunological techniques, which present several limitations, such as the inability to discriminate *Toxocara* species and cross-reactivity with other helminths.^3^ These limitations exacerbate the gap in knowledge regarding the pathology and prevalence of toxocariasis, consequently diminishing the prospects for effective treatment and the establishment of public health policies.^4^

Researchers have made significant efforts to develop innovative approaches to the diagnosis of infectious diseases. Bioinformatic tools are widely used for molecular biology and proteomics, including epitope-based vaccines, drug discovery, and diagnosis.^5^ ^,^ ^6^ ^,^ ^7^ Epitope mapping has gained attention as a major area of study. Through peptide prediction, T or B-cell epitopes are selected and synthesised through biological or chemical methods. Thus, peptide prediction facilitates the identification of these potential sites to replace the native antigen with the synthetic segment.^5^ ^,^ ^6^ ^,^ ^7^

Few studies have explored the use of synthetic epitopes for parasite diagnosis, including *Echinococcus* sp., *Schistosoma mansoni*, and *Strongyloides stercoralis*.^8^ ^-^ ^13^ Several innovative approaches are being evaluated to refine *Toxocara* diagnosis, yet the determination of synthetic epitopes for toxocariasis immunodiagnostics’ is unknown.^14^ ^-^ ^20^ Considering the lack of studies that have used *in silico* and *in vivo* epitope mapping as an innovative approach for *Toxocara* diagnosis, the present study aimed to identify, *in silico and in vivo*, potential epitopes from Tc-CTL-1, a TES-Ag protein, to improve the diagnostic methods for toxocariasis.

## MATERIALS AND METHODS

*Parasites* - Cercariae of *S. mansoni*, strain BH, were obtained through the experimental cycle maintained in *Biomphalaria glabrata* and hamsters (*Mesocricetus auratus*) respectively. The experimental cycle was carried out and maintained at Laboratório de Investigação Médica de Imunopatologia da Esquistossomose e outras Parasitoses do Hospital das Clínicas da Faculdade de Medicina da Universidade de São Paulo (LIM-06).^21^

*Ascaris suum* and *Toxocara canis* adult worms were obtained according to Leal-Silva et al.^22^ and Oliveira et al.^23^ Eggs were processed as described previously.^22^ ^,^ ^23^ *Strongyloides venezuelensis* filariform larvae were obtained according to Gouveia-Eufrasio et al.^24^

*Animals* - BALB/C and C57BL/6 mice were used for experiments with *T. canis*, *A. suum*, and *S. venezuelensis* as described by Leal-Silva et al.,^22^ Oliveira et al.^23^ and Gouveia-Eufrasio et al.,^24^ respectively. The animals were obtained from the Bioterium Facility of the Universidade Federal de Minas Gerais (UFMG), where the experiment was held.

BALB/c specific pathogen free (SPF) mice were used for experiments with *S. mansoni*. The animals were obtained from the Bioterium Facility of the Faculdade de Medicina of the Universidade de São Paulo (FMUSP).

All animals, from both facilities, were kept in clean cages, with food and water *ad libitum* and a 12/12 h light-dark cycle. Female C57BL/6 mice, aged eight weeks old, were inoculated orally with 2,500 embryonated *A. suum* eggs in phosphate-buffered saline (PBS) through a gavage needle as described by Oliveira et al.^23^ Female BALB/c mice aged six-eight weeks were inoculated subcutaneously with 700 *S. venezuelensis* filariform larvae.^24^ For *S. mansoni* infection, male BALB/c mice, aged six-eight weeks old, were inoculated with 60 cercariae. All animals were euthanised with lethal injections of 30 mg/kg xylazine and 300 mg/kg ketamine. Animal studies were approved by the Ethics Committee on Animal Use (CEUA) from the Universidade Federal de Minas Gerais (CEUA/UFMG, n. certificate #369/2018 and #200/2018), and Faculdade de Medicina da Universidade de São Paulo (n. certificate #393A and #1173/2018). All experiments followed the Ethical Guidelines of the National Council for the Control of Animal Experimentation.

*Sera* - Sera from *S. mansoni-*infected animals were collected through cardiac puncture after confirming heavy anaesthesia. Sera from animals experimentally infected with *A. suum*, *S. venezuelensis*, and *T. canis* were processed at the Immunobiology and Parasite Control Laboratory at UFMG, according to Oliveira et al.^23^ and Gouveia-Eufrasio et al.^24^ The samples were separated into aliquots and stored in a -20ºC freezer until processed.

*Toxocara canis protein sequence* - The Tc-CTL-1 protein sequence was obtained from the National Centre for Biotechnology Information (NCBI) protein database under the accession number AAB96779.1.

**Fig. 1:**
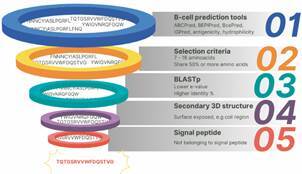
B-cell peptide prediction and selection criteria. Five bioinformatic tools predicted B-cell peptides (1). Selection criteria included, respectively, peptides predicted by at least two prediction tools and 50% or more shared aminoacid residues (2); homology with the Tc-CTL-1 (3); analysis based on secondary structure (4) and signal peptide (5).

*In silico analysis of the Tc-CTL-1 protein* - *In silico* analysis was carried out using ABCPred (recurrent neural network), Bepipred (neural network), BCEpred (physicochemical properties), and IgPRED (machine learning and physicochemical properties), based on the prediction of B-cell linear peptides.^5^ ^,^ ^6^ ^,^ ^7^ ^,^ ^25^ In addition, the Immune Epitope Database analysed B-cell peptide prediction based on physicochemical properties of hydrophilicity and antigenicity.^26^ ^,^ ^27^ SignalP 6.0 (https://services.healthtech.dtu.dk/service.php?SignalP) was used to confirm the signal peptide sequence and cleavage site. The Protein BLAST program (BLASTp) analysed the homology between the predicted sequences and proteins from the main databases. The sequences were evaluated, in order of importance, according to expected value (e-value) and percentage of identity. I-TASSER predicted the secondary structure of the protein, which was modelled using Pymol software (Schrödinger).^28^ Peptide sequences were analysed based on the primary and secondary structure of the three-dimensional model (Fig. 1). The selection criteria included: (1) sequences between 7 and 16 amino acids; (2) peptides predicted by at least two of the B-cell prediction tools; (3) peptide sequence that shared at least 50% of its amino acids; (4) Sequences with the lowest e-value and highest percentage of identity; (5) Peptides with the greatest number of amino acids residues in regions exposed to solvents in the secondary structure, such as coil; and (6) Sequences not belonging to the signal peptide.

*Peptide synthesis* - The peptides were synthesised in duplicates using the SPOT-synthesis technique on a cellulose membrane, according to Frank.^29^ This technique consists of the synthesis of solid phase peptides (SPPS) with the 9-fluorenylmethyloxycarbonyl group (Fmoc), with several spots on a membrane, in an automatic ResPep SL synthesiser (IntavisTM), following the distribution created by the synthesiser program. Free hydroxyl ends of the membrane served as anchoring points for the synthesis. Ethylene glycol units were coupled to the free ends of the amino acid, to intensify the stability of the peptide coupling to the membrane. The synthesis began with the C-terminal end of the last amino acid residue of the peptide, protected with Fmoc. Amino acids were activated with diisopropylcarbodiimide (DIC) at 1.1 M, and deposited on the membrane in two coupling cycles. Free amide ends (-NH2) were blocked with 3% acetic anhydride to avoid reactivity with side chains or unwanted bonds. Then, the Fmoc group was removed with 25% v/v piperidine and 4-methyl-piperidine in dimethylformamide (DMF). The cycle was repeated with all amino acids. At the end of the reaction, the side chain protections were removed with 95% (v/v) trichloroacetic acid, 2.5% (v/v) water, and 2.5% (v/v) triisopropylsilane. The membrane was washed four times with dichloromethane, four times with DMF, and finally, four times with ethanol. After drying, the presence of stains was verified under ultraviolet light.

*Dot-blot* - The dot-blot was performed according to Siqueira et al.^12^ Briefly, the cellulose membrane was blocked with a solution of 5% bovine serum albumin (BSA) and 4% sucrose in PBS, at room temperature and under moderate orbital agitation for 18 h. The membrane was washed three times, for 10 min each, in 0.1% PBS-Tween 20 (PBST). Pools of serum samples from all groups diluted 1:500 in PBST, were incubated for two hours at room temperature under rapid orbital shaking. After repeating the washing process, the membrane was incubated with peroxidase total mouse IgG (y-Chain specific; Sigma-Aldrich), diluted 1:10,000 in 0.1% PBST, for 1 h at room temperature in rapid orbital shaking. After a new washing cycle, the spots were determined using chemiluminescence (Amersham ECL prime western-blotting detection reagent). The spot signal intensities analyses were performed using ImageQuant LAS 4000 digital Imaging System Software. The membrane regeneration was conducted as described previously by Siqueira et al.^12^

*Scanning of spot signal intensities* - Densitometric analyses were performed according to Siqueira et al.^12^ using ImageJ software and the Protein Array Analyzer plugin (http://image.bio.methods.free.fr/ImageJ/?Protein-Array-Analyzer-for-ImageJ.html).

The densitometric analysis supports a better visual comparison, normalises the colours of the spot with the highest value, and allows a comparison between them. The densitometry values of each spot were calculated using colour intensity. The closer to the colour white, the greater the binding intensity with the peptide. The peptides were selected based on their specificity and reactivity. The background colour of the membrane was removed by calculating the mean of the four least reactive spots, and the value was subtracted from each spot in the membrane.

The densitometric ratio values were calculated to evaluate the reactivity of serum samples to Tc-CTL-1 peptides. Densitometric values were normalised based on the ratio of serum binding reactivity from animals inoculated with *T. canis* to the values of serum binding reactivity from non-infected animals. The same calculation was used for the densitometric values of sera from animals inoculated with other parasites. Peptides that presented values above 1.0 for the *T. canis*/non-infected, *T. canis*/*A. suum*, *T. canis*/*S. venezuelensis*, and *T. canis*/*S. mansoni* ratio were considered potential peptides. Selection criteria included the higher values from *T. canis*/non-infected ratio, followed by *T. canis*/*A. suum* ratio, *T. canis*/*S. mansoni*, and lastly, *T. canis*/*S. venezuelensis* ratio.

## RESULTS

*In silico analysis and B-cell prediction* - A total of fifty-five linear peptides were predicted and analysed for their amino acid sequence. These peptides range from 12 to 16 amino acids (Table I). The BLASTp analysis revealed a low e-value and high percentage of identity with the Tc-CTL-1 protein of *T. canis* [Supplementary data (Table I)]. There was a 100% homology with proteins from the same parasite such as collectin 12 and proteoglycans. There was no similarity with proteins from *Strongyloides* spp., *S. mansoni*, *A. suum*, or *Ascaris lumbricoides* [Supplementary data (Table I)].

**TABLE I:** B-cell predicted peptides from Tc-CTL-1, according to the membrane spots position

Peptide	Linear sequence
C1-L1	APPPAATTTAAPGVTT
C1-L2	TQTGSRVVWFDQSTVG
C1-L3	VFTNGSPVIFSNWRPS
C1-L4	NFLGQWDDAPCGSLFT
C1-L5	YWIGVNRQFGQW
C1-L6	SDWCTQTGSRVVWFDQ
C1-L7	GVTTTRPRACPPNWTP
C1-L8	APGVTTTRPRACPPN
C1-L9	TTAAPGVTTTRPRAC
C1-L10	PGVTTTRPRACPPNWT
C1-L11	NGSPVIFSNWRPSQPD
C1-L12	RGVTRYWIGVNRQFGQ
C1-L13	GVTRYWIGVNRQFGQ
C1-L14	DWCTQTGSRVVWFDQS
C1-L15	NQASDWCTQTGSRVVW
C1-L16	FLFNQASDWCTQTGSR
C1-L17	LGQWDDAPCGSLFTTP
C1-L18	FNQASDWCTQTGSRVV
C1-19	PAATTTAAPGVTTTRPRA
C2-L1	QSTVGNFGSELNFVNS
C2-L2	FNNNCYIASLPGRFL
C2-L3	VTRYWIGVNRQFGQWV
C2-L4	TRYWIGVNRQFGQWV
C2-L5	QWVFTNGSPVIFSNWR
C2-L6	PGRFLFNQASDWCTQT
C2-L7	TRYWIGVNRQFGQWVF
C2-L8	QASDWCTQTGSRVVWF
C2-L9	ASDWCTQTGSRVVWFD
C2-L10	PGRFLFNQASDWCTQT
C2-L11	RQFGQWVFTNGSPVIF
C2-L12	NNCYIASLPGRFLFNQ
C2-L13	RVVWFDQSTVGNFGSE
C2-L14	SNVTCAFVNYANFLGQ
C2-L15	RPRACPPNWTPFNNNC
C2-L16	SNWRPSQPDGCCGSNV
C2-L17	VNNVCVANNQGCNPPC
C2-L18	NPPCVAPQVCVAPMCV
C2-L19	RACPPNWTPFNNNCYI
C3-L1	VCVNNVCVANNQGCNP
C3-L2	GIFQVCVNNVCVANNQ
C3-L3	PSQPDGCCGSNVTCAF
C3-L4	QGCNPPCVAPQVCVAP
C3-L5	GSPVIFSNWRPSQPDG
C3-L6	CVNNVCVANNQGCNP
C3-L7	IFQVCVNNVCVANNQG
C3-L8	GSNVTCAFVNYANF
C3-L9	GRGVTRYWIGVNRQFG
C3-L10	GVTTTRPRACPPNWT
C3-L11	TNGSPVIFSNWRPSQP
C3-L12	IFQVCVNNVCVA
C3-L13	PAATTTAAPGVTTT
C3-L15	IFSNWRPSQPDGCCGS
C3-L16	GVTRYWIGVNRQFGQW
C4-L17	GSPVIFSNWRPSQPDG
C5-L16	CATNNDCGIFQVCVNN

**Fig. 2:**
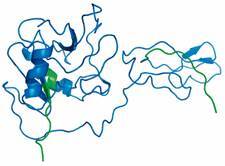
selected B-cell predicted peptide in the 3D structure of TC-CTL-1. Selected peptides are in blue.

**Fig. 3:**
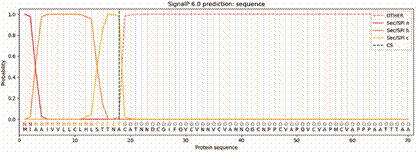
peptide signal sequence from Tc-CTL-1. SEC/SPI n - N-terminal region; SEC/SPI h - hydrophobic aminoacid residues; SEc/SPI c - the C-terminal portion; CS - cleavage site; *OTHER* - region predicted as non-signal peptide.

Analysis of the Tc-CTL-1 secondary structure revealed that peptides had at least two residues in coil regions, and 34 had at least one residue in alpha-helices or beta-sheets (Fig. 2). The SignalP 6.0 tool confirmed the presence of a signal peptide from the first to the 18th amino acid (Fig. 3).

**Fig. 4:**
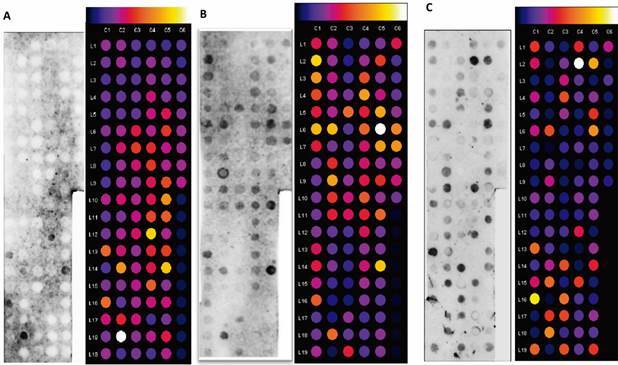
dot-blot from selected peptides against antibodies from: (A) serum of non-infected animals; (B) *Toxocara canis*-infected animals; and (C) *Ascaris suum*-infected animals. The colourimetric scale on the top of the image shows the intensity of reactivity, ranging from non-reagent (black) to very reagent (white). Spots from C3-L17 to C6-L9 are duplicates, except for those added to Table I (spot C3-L14 is empty).

**Fig. 5:**
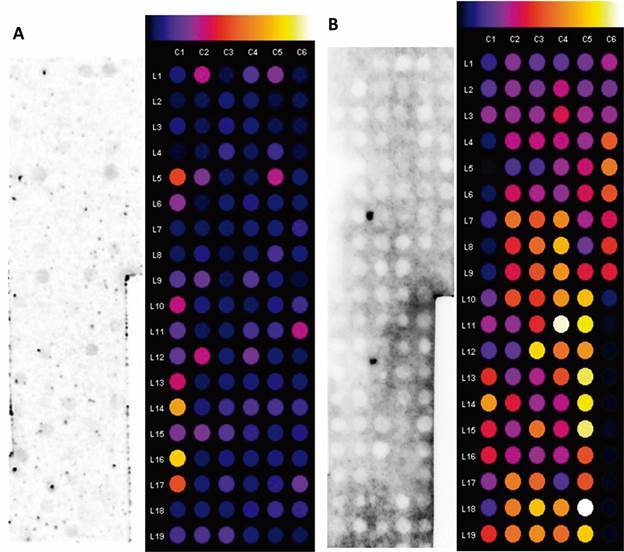
dot-blot from selected peptides against antibodies from sera of (A) *Strongyloides venezuelensis*-infected animals; (B) and *Schistosoma mansoni*-infected animals. The colorimetric scale on the top of the image shows the intensity of reactivity, ranging from non-reagent (black) to very reagent (white). Spots from C3-L17 to C6-L9 are duplicates, except for those added to Table I (spot C3-L14 is empty).

*Dot-blot and peptide selection* - The dot-blot results showed antibody recognition of the selected peptides. *A. suum* and *T. canis* serum recognised the greatest number of peptides [Fig. 4B-C, Supplementary data (Tables III-IV)]. Non-infected control serum, *S. venezuelensis* serum, and *S. mansoni* serum were able to recognise a few peptides [Figs 4A, 5A-B, Supplementary data (Tables II, V, VI)]. Antibody recognition was not consistent across duplicates.

The ratios between densitometric values of sera from animals infected with T.*canis*, non-infected, and infected with other parasites showed that almost all peptides are potential epitopes depending on the group studied [Supplementary data (Tables VII, VIII, IX, X)]. The results revealed eight potential epitopes based on *T. canis*/non-infected ratio; 29 potential epitopes based on *T. canis*/*A. suum* ratio; 49 potential epitopes based on *T. canis*/*S. venezuelensis* ratio; and 11 potential epitopes based on *T. canis*/*S. mansoni* ratio [Table II, Supplementary data (Tables VII, VIII, IX, X)].

**TABLE II:** Densitometric ratio values of spots reactivity from infected and non-infected animal sera. Peptide values above one is in blue and bold and indicates a potential B-cell epitope. Duplicate spots were removed for better visualisation

	Densitometric ratio values											
	*Toxocara canis* / non-infected			*T. canis* / *Ascaris suum*			*T. canis* / *Strongyloides venezuelensis*			*T. canis /* *Schistosoma mansoni*		
	L1	L2	L3	L1	L2	L3	L1	L2	L3	L1	L2	L3
C1	0,5	0,4	0,2	0,6	1,4	0,8	6,1	1,4	3,3	1,8	0,7	0,2
C2	1,7	0,6	0,2	1,4	4,3	0,3	30,0	8,9	1,9	2,4	0,6	0,3
C3	1,5	0,6	0,3	6,2	2,7	0,3	9,9	9,7	2,3	1,4	0,7	0,3
C4	1,2	0,7	0,5	1,0	2,2	0,3	25,5	10,9	2,6	4,5	0,6	0,4
C5	1,3	0,6	1,1	1,7	4,6	1,4	1,3	1,8	14,2	24,5	1,1	2,1
C6	1,4	1,3	0,4	1,5	1,0	1,6	4,1	31,7	3,7	7,9	1,2	0,4
C7	0,9	0,5	0,4	3,2	5,9	1,1	9,6	6,0	4,9	1,9	0,2	0,2
C8	0,4	0,8	0,4	1,6	1,4	2,0	5,9	6,4	7,9	3,3	0,6	0,2
C9	0,4	1,3	0,5	1,9	1,4	3,4	1,8	4,6	8,7	1,7	1,0	0,3
C10	0,3	0,7	0,6	0,6	1,3	2,5	1,1	7,1	6,6	0,6	0,5	0,4
C11	0,4	0,6	0,5	1,1	2,1	2,6	1,8	11,6	8,4	0,5	0,9	0,5
C12	0,5	0,3	0,3	1,4	0,7	0,7	1,8	1,0	3,0	0,9	0,6	0,1
C13	0,5	0,3	0,2	0,5	0,8	1,4	1,6	5,8	2,5	0,5	0,5	0,3
C14	0,7	0,2	0,2	1,7	0,5	0,2	0,9	3,9	1,4	0,4	0,4	0,3
C15	0,4	0,2	0,1	0,6	0,2	0,8	2,0	0,8	0,9	0,5	0,3	0,1
C16	0,4	0,2	0,1	0,4	2,1	0,1	1,0	2,4	1,2	0,8	0,2	0,2
C17	0,3	0,1	0,4	2,3	0,1	0,4	0,6	1,8	2,6	0,4	0,1	0,3
C18	0,5	0,3	0,4	2,6	0,6	1,1	4,7	10,5	4,5	0,8	0,6	0,2
C19	0,5	0,1	0,8	0,4	0,6	0,6	2,9	0,5	3,5	0,4	0,1	0,4

The selection criteria narrowed to four potential epitopes: C1-L2, C1-L3, C1-L5, and C1-L6.

## DISCUSSION

The limitations of immunodiagnosis of toxocariasis hinder the ability to track infection occurrence, affecting clinical management and treatment of affected individuals. The gaps in epidemiological knowledge caused by those limitations prevent the implementation of strategies by public health programs.^3^ ^,^ ^30^ ^,^ ^31^

As a result of the combination of physicochemical properties and machine learning methods, it was possible to predict B-cell linear peptides from Tc-CTL-1 protein, including peptides that have up to 16 amino acids and shared 50% or more amino acids. Additionally, we evaluated selected peptides according to their secondary structure in association with physicochemical scales, with preference given to peptides in the coil region. This approach was chosen as solvent-exposed peptides have been demonstrated to have a higher propensity to elicit an immune response.^25^ Among the peptides selected *in silico*, the present study identified those with amino acids between positions 107 and 158 as exhibiting the most promising immunogenic results by dot-blot.

Only one previous study analysed, *in silico*, TES-Ag peptides for diagnostic purposes, evaluating the predicted peptides from the most studied proteins in the literature and proposing a chimeric protein formed by peptides from lectins and mucins connected by a linker.^15^ Despite Tc-CTL-1’s high homology with mammalian C-type lectins and its demonstrated immunogenicity across numerous diagnostic methods, there is a lack of experimental data evaluating the potential epitopes of this protein.^32^ ^,^ ^33^ ^,^ ^34^ ^,^ ^35^ ^,^ ^36^ Peptides from TES-Ag proteins that have been studied by other researchers have focused on the carbohydrate and glycan-binding portions.^37^ ^,^ ^38^ ^,^ ^39^ ^,^ ^40^ ^,^ ^41^ The study showed that chemically synthesised glycans from the excretory-secretory antigens are recognised by anti-*Toxocara* antibodies. However, the synthesis technique employed was not the same as that described in the present study.

Performing the peptide synthesis directly on the membrane, with an anchoring point in cellulose, decreases the chances of hiding immunogenic sites, usually from changes in conformation. In addition, the reusable membrane allowed the repetition of the experiment, which confirmed its reproducibility and the reactivity of the epitope by the antibodies. Nevertheless, some results were not reproducible. On the colourimetric scale, contradictory results were observed between duplicates C1-L3 and C3-L19. Unevenness in the container or agitator base may be the cause of this result, as it may expose one part of the membrane to the samples more than the other. Therefore, a peptide was considered a reagent when at least one replicate followed the selection criteria for dot-blot.

The ratio values were the main selection criteria. When analysing the results of the infected group, there was a priority to include epitopes with greater potential for discrimination between *Toxocara* and *Ascaris* due to their phylogenetic proximity and intense cross-reactivity.^42^ ^,^ ^43^ The second priority was peptides that differentiate between *Toxocara* and *S. mansoni*, as the latter showed some cross-reactivity with Tc-CTL-1 peptides on the membrane. The cross-reactivity with *A. suum* samples might have been the result of the inoculation with a higher burden. If the inoculum changes, results might be like those observed in *Toxocara*-infected animals.^44^ Sera from animals infected with *S. venezuelensis* recognised fewer peptides at a lower intensity, indicating the high specificity of Tc-CTL-1. It would be interesting to investigate how anti-*Toxocara* antibodies bind to each segment of the Tc-CTL-1 protein during the early and late stages of infection, as known with TES-Ag, as well as its cross-reactivity with *Ascaris* spp.^44^ ^,^ ^45^ ^,^ ^46^ ^,^ ^47^ ^,^ ^48^

Although BLASTp results did not show homology with proteins from other parasites, sera from evaluated animals inoculated with *A. suum* and non-infected controls showed cross-reactivity with several spots in the membrane. This may have occurred due to the lack of protein sequences from *Toxocara*, *Ascaris*, and other helminths submitted in the main protein databases, hampering the homology evaluation between them. In studies with *Leishmania* and *Echinococcus*, the experimental results corroborated the homology analysis.^8^ ^,^ ^12^ ^,^ ^49^ These data emphasised the need for stimulating further research on helminths so that diagnostic improvement of toxocariasis is feasible.

Analysis of nonspecific spots and spots with high cross-reactivity revealed that antibodies from all groups recognised epitopes that shared a smaller amino acid sequence. The amino acid residues DWCTQTGSR were identified as being present in both the C1-L6 and C1-L5 peptides. This finding suggests that a single parameter may not be adequate for evaluating peptide immunogenicity. Evaluating the immunogenicity of peptides on the colourimetric scale without applying ratio calculation and data normalisation might underestimate the immunogenic potential of some sequences. Furthermore, cross-reactive amino acid residues belonging to a potentially immunogenic epitope may not be the binding site for anti-*Toxocara* IgG, which could bind to the remainder of the sequence. Alternatively, the difference in intensity of cross-reactivity of peptides may happened because of how the amino acid residues were arranged to form the peptide, especially those with common non-specific amino acids. In this sense, further research is needed to evaluate these hypotheses.

The main limitation of this study was the volume of serum, the SPOT synthesis technique, and the prediction tools used. Mice sera are scarce due to their size, and so, analysis with individual samples with the SPOT-synthesis technique becomes impractical. The SPOT-synthesis technique is mainly for peptide screening, which also limits the analysis of individual samples.

The findings of the present study indicate that synthetic peptides represent a promising alternative for enhancing the diagnosis of toxocariasis by identifying four potentially immunogenic epitopes from the Tc-CTL-1 protein. The reactivity of Tc-CTL-1 peptides observed in the SPOT synthesis broadens the scope of research possibilities for epitope-based studies with other TES-Ag proteins. The dot-blot results obtained through SPOT synthesis indicate the potential for adapting this technique for the detection of single epitopes for diagnostic purposes. In conclusion, the results underscore the relevance of proteomic investigations with other *Toxocara* species and other helminths to facilitate the advancement of our understanding of this zoonosis.
