# Supplementary material for: Immunogenic mapping of potential epitopes from Tc-CTL-1 for the diagnosis of murine toxocariasis
Source: Mem Inst Oswaldo Cruz. 2025 Mar 14;120:e240111. doi: 10.1590/0074-02760240111 (PMC11908769; doi:10.1590/0074-02760240111)
Supplement: Supplementary file 1 [file 1678-8060-mioc-120-e240111-s.pdf]

TABLE I

BLASTp homology results on selected peptides. The homology results displayed are only for Tc-CTL-1 protein

Sequence	Description	Scientific name	Common name	Taxid	Max. score	Total score	Total cover	E- value	Percent ident.
IFSNWRPSQPDGCCGS	excretory/secretory C-type lectin TES-32 [Toxocara canis]	*Toxocara canis*	dog roundworm	6265	41.4	41.4	100%	9,00E-03	100.00
QWVFTNGSPVIFSNWRPSQPDG	excretory/secretory C-type lectin TES-32 [Toxocara canis]	*Toxocara canis*	dog roundworm	6265	77.4	77.4	100%	1,00E-14	100.00
RPRACPPNWTPFNNNCYIAS	excretory/secretory C-type lectin TES-32 [Toxocara canis]	*Toxocara canis*	dog roundworm	6265	73.2	73.2	100%	3,00E-13	100.00
SNWRPSQPDGCCGSNVTCAF	excretory/secretory C-type lectin TES-32 [Toxocara canis]	*Toxocara canis*	dog roundworm	6265	70.2	70.2	100%	3,00E-12	100.00
VNNVCVANNQGCNPPCVAPQ	excretory/secretory C-type lectin TES-32 [Toxocara canis]	*Toxocara canis*	dog roundworm	6265	69.4	69.4	100%	6,00E-12	100.00
PGRFLFNQASDWCTQTGSRV	excretory/secretory C-type lectin TES-32 [Toxocara canis]	*Toxocara canis*	dog roundworm	6265	68.9	68.9	100%	9,00E-12	100.00
NPPCVAPQVCVAPMCVAPP	excretory/secretory C-type lectin TES-32 [Toxocara canis]	*Toxocara canis*	dog roundworm	6265	67.2	67.2	100%	3,00E-11	100.00
NFLGQWDDAPCGSLFTTPQ	excretory/secretory C-type lectin TES-32 [Toxocara canis]	*Toxocara canis*	dog roundworm	6265	66.4	66.4	100%	6,00E-11	100.00
RACPPNWTPFNNNCYI	excretory/secretory C-type lectin TES-32 [Toxocara canis]	*Toxocara canis*	dog roundworm	6265	61.3	61.3	100%	2,00E-09	100.00
TRYWIGVNRQFGQWVF	excretory/secretory C-type lectin TES-32 [Toxocara canis]	*Toxocara canis*	dog roundworm	6265	60.4	60.4	100%	4,00E-09	100.00
VTRYWIGVNRQFGQWV	excretory/secretory C-type lectin TES-32 [Toxocara canis]	*Toxocara canis*	dog roundworm	6265	59.6	59.6	100%	8,00E-09	100.00
GVTRYWIGVNRQFGQW	excretory/secretory C-type lectin TES-32 [Toxocara canis]	*Toxocara canis*	dog roundworm	6265	59.2	59.2	100%	1,00E-08	100.00
SDWCTQTGSRVVWFDQ	excretory/secretory C-type lectin TES-32 [Toxocara canis]	*Toxocara canis*	dog roundworm	6265	58.7	58.7	100%	2,00E-08	100.00
DWCTQTGSRVVWFDQS	excretory/secretory C-type lectin TES-32 [Toxocara canis]	*Toxocara canis*	dog roundworm	6265	58.7	58.7	100%	2,00E-08	100.00
QASDWCTQTGSRVVWF	excretory/secretory C-type lectin TES-32 [Toxocara canis]	*Toxocara canis*	dog roundworm	6265	57.9	57.9	100%	3,00E-08	100.00
ASDWCTQTGSRVVWFD	excretory/secretory C-type lectin TES-32 [Toxocara canis]	*Toxocara canis*	dog roundworm	6265	57.9	57.9	100%	3,00E-08	100.00
NQASDWCTQTGSRVVW	excretory/secretory C-type lectin TES-32 [Toxocara canis]	*Toxocara canis*	dog roundworm	6265	57.5	57.5	100%	5,00E-08	100.00
PGRFLFNQASDWCTQT	excretory/secretory C-type lectin TES-32 [Toxocara canis]	*Toxocara canis*	dog roundworm	6265	57.5	57.5	100%	5,00E-08	100.00
CATNNDCGIFQVCVNN	excretory/secretory C-type lectin TES-32 [Toxocara canis]	*Toxocara canis*	dog roundworm	6265	57.5	57.5	100%	5,00E-08	100.00
RGVTRYWIGVNRQFGQ	excretory/secretory C-type lectin TES-32 [Toxocara canis]	*Toxocara canis*	dog roundworm	6265	57.1	57.1	100%	6,00E-08	100.00
RQFGQWVFTNGSPVIF	excretory/secretory C-type lectin TES-32 [Toxocara canis]	*Toxocara canis*	dog roundworm	6265	57.1	57.1	100%	6,00E-08	100.00
TRYWIGVNRQFGQWV	excretory/secretory C-type lectin TES-32 [Toxocara canis]	*Toxocara canis*	dog roundworm	6265	56.6	56.6	100%	8,00E-08	100.00
GVTTTRPRACPPNWTP	excretory/secretory C-type lectin TES-32 [Toxocara canis]	*Toxocara canis*	dog roundworm	6265	56.6	56.6	100%	9,00E-08	100.00
PGVTTTRPRACPPNWT	excretory/secretory C-type lectin TES-32 [Toxocara canis]	*Toxocara canis*	dog roundworm	6265	56.6	56.6	100%	9,00E-08	100.00
FLFNQASDWCTQTGSR	excretory/secretory C-type lectin TES-32 [Toxocara canis]	*Toxocara canis*	dog roundworm	6265	56.6	56.6	100%	9,00E-08	100.00
NNCYIASLPGRFLFNQ	excretory/secretory C-type lectin TES-32 [Toxocara canis]	*Toxocara canis*	dog roundworm	6265	56.6	56.6	100%	9,00E-08	100.00
VCVNNVCVANNQGCNP	excretory/secretory C-type lectin TES-32 [Toxocara canis]	*Toxocara canis*	dog roundworm	6265	56.6	56.6	100%	9,00E-08	100.00
NGSPVIFSNWRPSQPD	excretory/secretory C-type lectin TES-32 [Toxocara canis]	*Toxocara canis*	dog roundworm	6265	56.2	56.2	100%	1,00E-07	100.00
GIFQVCVNNVCVANNQ	excretory/secretory C-type lectin TES-32 [Toxocara canis]	*Toxocara canis*	dog roundworm	6265	56.2	56.2	100%	1,00E-07	100.00
QGCNPPCVAPQVCVAP	excretory/secretory C-type lectin TES-32 [Toxocara canis]	*Toxocara canis*	dog roundworm	6265	56.2	56.2	100%	1,00E-07	100.00
GRGVTRYWIGVNRQFG	excretory/secretory C-type lectin TES-32 [Toxocara canis]	*Toxocara canis*	dog roundworm	6265	56.2	56.2	100%	1,00E-07	100.00
VFTNGSPVIFSNWRPS	excretory/secretory C-type lectin TES-32 [Toxocara canis]	*Toxocara canis*	dog roundworm	6265	55.8	55.8	100%	2,00E-07	100.00
LGQWDDAPCGSLFTTP	excretory/secretory C-type lectin TES-32 [Toxocara canis]	*Toxocara canis*	dog roundworm	6265	55.8	55.8	100%	2,00E-07	100.00
FNQASDWCTQTGSRVV	excretory/secretory C-type lectin TES-32 [Toxocara canis]	*Toxocara canis*	dog roundworm	6265	55.8	55.8	100%	2,00E-07	100.00
PAATTTAAPGVTTTRPRA	excretory/secretory C-type lectin TES-32 [Toxocara canis]	*Toxocara canis*	dog roundworm	6265	56.2	56.2	100%	2,00E-07	100.00
TNGSPVIFSNWRPSQP	excretory/secretory C-type lectin TES-32 [Toxocara canis]	*Toxocara canis*	dog roundworm	6265	55.8	55.8	100%	2,00E-07	100.00
GSPVIFSNWRPSQPDG	excretory/secretory C-type lectin TES-32 [Toxocara canis]	*Toxocara canis*	dog roundworm	6265	55.4	55.4	100%	3,00E-07	100.00
RVVWFDQSTVGNFGSE	excretory/secretory C-type lectin TES-32 [Toxocara canis]	*Toxocara canis*	dog roundworm	6265	55.4	55.4	100%	3,00E-07	100.00
PSQPDGCCGSNVTCAF	excretory/secretory C-type lectin TES-32 [Toxocara canis]	*Toxocara canis*	dog roundworm	6265	55.4	55.4	100%	3,00E-07	100.00
GSPVIFSNWRPSQPDG	excretory/secretory C-type lectin TES-32 [Toxocara canis]	*Toxocara canis*	dog roundworm	6265	55.4	55.4	100%	3,00E-07	100.00
SNVTCAFVNYANFLGQ	excretory/secretory C-type lectin TES-32 [Toxocara canis]	*Toxocara canis*	dog roundworm	6265	54.9	54.9	100%	4,00E-07	100.00
TQTGSRVVWFDQSTVG	excretory/secretory C-type lectin TES-32 [Toxocara canis]	*Toxocara canis*	dog roundworm	6265	54.1	54.1	100%	7,00E-07	100.00
CVNNVCVANNQGCNP	excretory/secretory C-type lectin TES-32 [Toxocara canis]	*Toxocara canis*	dog roundworm	6265	53.7	53.7	100%	8,00E-07	100.00
IFQVCVNNVCVANNQG	excretory/secretory C-type lectin TES-32 [Toxocara canis]	*Toxocara canis*	dog roundworm	6265	53.7	53.7	100%	8,00E-07	100.00
GVTRYWIGVNRQFGQ	excretory/secretory C-type lectin TES-32 [Toxocara canis]	*Toxocara canis*	dog roundworm	6265	53.7	53.7	100%	9,00E-07	100.00
FNNNCYIASLPGRFL	excretory/secretory C-type lectin TES-32 [Toxocara canis]	*Toxocara canis*	dog roundworm	6265	53.2	53.2	100%	1,00E-06	100.00
GVTTTRPRACPPNWT	excretory/secretory C-type lectin TES-32 [Toxocara canis]	*Toxocara canis*	dog roundworm	6265	53.2	53.2	100%	1,00E-06	100.00
QSTVGNFGSELNFVNS	excretory/secretory C-type lectin TES-32 [Toxocara canis]	*Toxocara canis*	dog roundworm	6265	52.4	52.4	100%	3,00E-06	100.00
APGVTTTRPRACPPN	excretory/secretory C-type lectin TES-32 [Toxocara canis]	*Toxocara canis*	dog roundworm	6265	50.7	50.7	100%	9,00E-06	100.00
APPPAATTTAAPGVTT	excretory/secretory C-type lectin TES-32 [Toxocara canis]	*Toxocara canis*	dog roundworm	6265	49.8	49.8	100%	2,00E-05	100.00
TTAAPGVTTTRPRAC	excretory/secretory C-type lectin TES-32 [Toxocara canis]	*Toxocara canis*	dog roundworm	6265	49.0	49.0	100%	4,00E-05	100.00
GSNVTCAFVNYANF	excretory/secretory C-type lectin TES-32 [Toxocara canis]	*Toxocara canis*	dog roundworm	6265	48.6	48.6	100%	4,00E-05	100.00
YWIGVNRQFGQW	excretory/secretory C-type lectin TES-32 [Toxocara canis]	*Toxocara canis*	dog roundworm	6265	47.3	47.3	100%	8,00E-05	100.00
IFQVCVNNVCVA	excretory/secretory C-type lectin TES-32 [Toxocara canis]	*Toxocara canis*	dog roundworm	6265	43.5	43.5	100%	2,00E-03	100.00
PAATTTAAPGVTTT	excretory/secretory C-type lectin TES-32 [Toxocara canis]	*Toxocara canis*	dog roundworm	6265	43.5	43.6	100%	3,00E-03	100.00

CC BY

TABLE II

Densitometric reactivity values from serum samples of non-infected animals to selected peptides

	Columns (C)					
Lines (L)	C1	C2	C3	C4	C5	C6
L1	23410	21703	12268	18420	12926	17824
L2	14469	14207	16333	19241	18127	15085
L3	12914	15624	14420	16189	13903	24251
L4	12438	13243	15098	15564	11616	24261
L5	9520	14907	16012	16866	21051	18635
L6	16084	17677	14695	16648	22933	16287
L7	13373	13502	16222	21050	18999	15267
L8	18759	17443	17032	20567	18298	21744
L9	19318	15746	18074	24767	30608	22152
L10	23124	21149	16938	26599	37564	
L11	17177	20198	22844	26377	31421	
L12	15414	21408	19745	31248	20773	
L13	22387	22136	18524	25995	30843	
L14	18366	32729	20823	20038	34725	
L15	22347	17768	25999	24523	16566	
L16	43178	14154	20569	18461	21418	
L17	19335	21139	19314	10880	15369	
L18	15026	56985	15296	15904	20879	
L19	18412	11547	16525	11521	17234	

TABLE III

Densitometric reactivity values from serum samples of *Toxocara canis*-infected animals to selected peptides

	Columns (C)					
Lines (L)	C1	C2	C3	C4	C5	C6
L1	23410	21703	12268	18420	12926	17824
L2	14469	14207	16333	19241	18127	15085
L3	12914	15624	14420	16189	13903	24251
L4	12438	13243	15098	15564	11616	24261
L5	9520	14907	16012	16866	21051	18635
L6	16084	17677	14695	16648	22933	16287
L7	13373	13502	16222	21050	18999	15267
L8	18759	17443	17032	20567	18298	21744
L9	19318	15746	18074	24767	30608	22152
L10	23124	21149	16938	26599	37564	
L11	17177	20198	22844	26377	31421	
L12	15414	21408	19745	31248	20773	
L13	22387	22136	18524	25995	30843	
L14	18366	32729	20823	20038	34725	
L15	22347	17768	25999	24523	16566	
L16	43178	14154	20569	18461	21418	
L17	19335	21139	19314	10880	15369	
L18	15026	56985	15296	15904	20879	
L19	18412	11547	16525	11521	17234	

TABLE IV

Densitometric reactivity values from serum samples of *Ascaris suum*-infected animals to selected peptides

	Columns (C)					
Lines (L)	C1	C2	C3	C4	C5	C6
L1	20518	6995	3298	19010	6487	14316
L2	16734	1950	11009	44981	31421	1760
L3	3217	3630	15364	8849	2559	8290
L4	14512	4172	22476	9826	11947	3015
L5	7292	2044	12680	3351	20302	1992
L6	14929	22275	3466	4796	29275	3468
L7	3944	1230	5865	9117	6510	2860
L8	4632	9321	3389	1955	6710	3558
L9	3661	14764	2456	7294	10624	4915
L10	11593	10817	4246	11912	9487	
L11	6621	6011	4399	5509	11730	
L12	5536	9188	7000	17898	2594	
L13	25287	7780	2962	1803	12428	
L14	7117	13990	22336	2353	18796	
L15	16407	24719	4326	20793	3563	
L16	37590	1473	25547	7518	4967	
L17	2539	20517	21753	7564	12518	
L18	2625	27977	6016	10556	8624	
L19	24064	2602	22469	9071	20384	

TABLE V

Densitometric reactivity values from serum samples of *Strongyloides venezuelensis*-infected animals to selected peptides

	Columns (C)					
Lines (L)	C1	C2	C3	C4	C5	C6
L1	1930	6547	815	3735	5211	875
L2	801	943	1854	1441	708	1082
L3	2015	1017	2138	1743	888	842
L4	591	853	2805	1102	2870	690
L5	9905	5098	1211	1159	6425	1515
L6	5468	709	1522	2150	1722	1297
L7	1300	1223	1297	1709	1694	2497
L8	1235	2063	862	1281	3209	1778
L9	3773	4528	950	3673	1857	
L10	6873	1946	1614	1247	2070	
L11	3938	1104	1379	3228	2978	
L12	4263	6901	1691	4944	1614	
L13	7224	1091	1730	1830	2071	
L14	13958	1944	2901	3434	3295	
L15	4971	5051	3712	2512	1659	
L16	15846	1249	2017	1745	2006	
L17	10270	1671	3224	1640	2351	
L18	1470	1632	1450	2172	2756	
L19	3111	3416	3680	1754	1246	

TABLE VI

Densitometric reactivity values from serum samples of *Schistosoma mansoni*-infected animals to selected peptides

	Columns (C)					
Lines (L)	C1	C2	C3	C4	C5	C6
L1	6466	13615	10852	10606	12344	15685
L2	9910	13736	12462	17295	12842	12159
L3	14193	14400	14087	20033	15225	14290
L4	3312	16813	17429	17306	14735	26864
L5	507	8667	8351	14780	18176	29942
L6	2824	18990	15704	14233	19586	25985
L7	6517	29591	26328	32140	15509	19691
L8	2201	22137	28812	37372	11710	23054
L9	3948	20368	25834	33541	21292	21555
L10	11745	25472	23604	33178	38186	
L11	15290	14121	22207	48331	43203	
L12	9155	10756	40842	29513	33542	
L13	22654	13391	15858	25621	44810	
L14	34069	21035	14879	16604	42711	
L15	20998	14919	29225	18420	45398	
L16	21039	16632	14762	15921	25944	
L17	13709	30860	27889	9176	26012	
L18	8291	30628	38703	33344	51651	
L19	22704	29643	32571	28703	40003	

TABLE VII

Toxocara canis/non-infected ratio values. Peptides with values above 1 are in bold

	Columns (C)					
Lines (L)	C1	C2	C3	C4	C5	C6
L1	0,5	0,4	0,2	0,3	0,6	0,7
L2	**1,7**	0,6	0,2	0,4	0,8	0,3
L3	**1,5**	0,6	0,3	0,9	0,6	0,2
L4	**1,2**	0,7	0,5	**1,3**	0,9	0,3
L5	**1,3**	0,6	1,1	0,8	1	0,4
L6	**1,4**	1,3	0,4	1	**1,3**	**1,2**
L7	0,9	0,5	0,4	0,5	**1,1**	**1,3**
L8	0,4	0,8	0,4	0,5	0,5	0,4
L9	0,4	**1,3**	0,5	0,5	0,4	0,5
L10	0,3	0,7	0,6	0,6	0,2	
L11	0,4	0,6	0,5	0,5	0,5	
L12	0,5	0,3	0,3	0,4	0,3	
L13	0,5	0,3	0,2	0,3	0,2	
L14	0,7	0,2	0,2	0,5	0,7	
L15	0,4	0,2	0,1	0,4	0,4	
L16	0,4	0,2	0,1	0,4	0,3	
L17	0,3	0,1	0,4	0,4	0,4	
L18	0,5	0,3	0,4	0,4	0,4	
L19	0,5	0,1	0,8	0,5	0,4	

TABLE VIII

Toxocara canis / *Ascaris suum* ratio values. Peptides with values above 1 are in bold

	Columns (C)					
Lines (L)	C1	C2	C3	C4	C5	C6
L1	0,6	**1,4**	0,8	0,3	**1,2**	0,8
L2	**1,4**	**4,3**	0,3	0,2	0,5	**2,9**
L3	**6,2**	**2,7**	0,3	**1,7**	**3,1**	0,6
L4	1	**2,2**	0,3	**2,1**	0,8	**2,2**
L5	**1,7**	**4,6**	**1,4**	**3,8**	1,1	**4,1**
L6	**1,5**	1	**1,6**	**3,5**	1	**5,5**
L7	**3,2**	**5,9**	1,1	**1,2**	**3,2**	**7**
L8	**1,6**	**1,4**	**2**	**5,7**	**1,3**	**2,5**
L9	**1,9**	**1,4**	**3,4**	**1,7**	**1,3**	**2,1**
L10	0,6	**1,3**	**2,5**	**1,3**	0,9	
L11	1,1	**2,1**	**2,6**	**2,3**	**1,4**	
L12	**1,4**	0,7	0,7	0,7	**2,7**	
L13	0,5	0,8	**1,4**	**4,6**	0,6	
L14	**1,7**	0,5	0,2	**4,5**	**1,2**	
L15	0,6	0,2	0,8	0,4	**2**	
L16	0,4	**2,1**	0,1	1	**1,2**	
L17	**2,3**	0,1	0,4	0,6	0,5	
L18	**2,6**	0,6	1,1	0,6	0,9	
L19	0,4	0,6	0,6	0,6	0,4	

TABLE IX

Toxocara canis / *Strongyloides venezuelensis* ratio values. Peptides with values above 1 are in bold

	Columns (C)					
Lines (L)	C1	C2	C3	C4	C5	C6
L1	**6,1**	**1,4**	**3,3**	**1,6**	**1,4**	**13,8**
L2	**30**	**8,9**	**1,9**	**6**	**20,2**	**4,7**
L3	**9,9**	**9,7**	**2,3**	**8,8**	**9,1**	**6,2**
L4	**25,5**	**10,9**	**2,6**	**18,7**	**3,5**	**9,4**
L5	**1,3**	**1,8**	**14,2**	**11**	**3,3**	**5,4**
L6	**4,1**	**31,7**	**3,7**	**7,8**	**17,8**	**14,7**
L7	**9,6**	**6**	**4,9**	**6,4**	**12,2**	**8**
L8	**5,9**	**6,4**	**7,9**	**8,7**	**2,7**	**5**
L9	**1,8**	**4,6**	**8,7**	**3,3**	**7,2**	
L10	1,1	**7,1**	**6,6**	**12,8**	**4,1**	
L11	**1,8**	**11,6**	**8,4**	**4**	**5,6**	
L12	**1,8**	1	**3**	**2,4**	**4,4**	
L13	**1,6**	**5,8**	**2,5**	**4,6**	**3,6**	
L14	0,9	**3,9**	**1,4**	**3,1**	**7,1**	
L15	**2**	0,8	0,9	**3,5**	**4,2**	
L16	1	**2,4**	**1,2**	**4,4**	**2,9**	
L17	0,6	**1,8**	**2,6**	**2,8**	**2,6**	
L18	**4,7**	**10,5**	**4,5**	**2,9**	**2,9**	
L19	**2,9**	0,5	**3,5**	**3,2**	**6,2**	

TABLE X

Toxocara canis / *Schistosoma mansoni* ratio values. Peptides with values above 1 are in bold

	Columns (C)					
Lines (L)	C1	C2	C3	C4	C5	C6
L1	**1,8**	0,7	0,2	0,6	0,6	0,8
L2	**2,4**	0,6	0,3	0,5	**1,1**	0,4
L3	**1,4**	0,7	0,3	0,8	0,5	0,4
L4	**4,5**	0,6	0,4	**1,2**	0,7	0,2
L5	**24,5**	1,1	**2,1**	0,9	**1,2**	0,3
L6	**7,9**	**1,2**	0,4	**1,2**	**1,6**	0,7
L7	**1,9**	0,2	0,2	0,3	**1,3**	1
L8	**3,3**	0,6	0,2	0,3	0,7	0,4
L9	**1,7**	1	0,3	0,4	0,6	0,5
L10	0,6	0,5	0,4	0,5	0,2	
L11	0,5	0,9	0,5	0,3	0,4	
L12	0,9	0,6	0,1	0,4	0,2	
L13	0,5	0,5	0,3	0,3	0,2	
L14	0,4	0,4	0,3	0,6	0,5	
L15	0,5	0,3	0,1	0,5	0,2	
L16	0,8	0,2	0,2	0,5	0,2	
L17	0,4	0,1	0,3	0,5	0,2	
L18	0,8	0,6	0,2	0,2	0,2	
L19	0,4	0,1	0,4	0,2	0,2	
